# Targeting B Cells and Plasma Cells in Autoimmune Diseases

**DOI:** 10.3389/fimmu.2018.00835

**Published:** 2018-04-23

**Authors:** Katharina Hofmann, Ann-Katrin Clauder, Rudolf Armin Manz

**Affiliations:** Institute for Systemic Inflammation Research, University of Luebeck, Luebeck, Schleswig-Holstein, Germany

**Keywords:** B cells, IL-10^+^ B cell, autoantibodies, autoimmune disease, rheumatoid arthritis, rituximab, bortezomib

## Abstract

Success with B cell depletion using rituximab has proven the concept that B lineage cells represent a valid target for the treatment of autoimmune diseases, and has promoted the development of other B cell targeting agents. Present data confirm that B cell depletion is beneficial in various autoimmune disorders and also show that it can worsen the disease course in some patients. These findings suggest that B lineage cells not only produce pathogenic autoantibodies, but also significantly contribute to the regulation of inflammation. In this review, we will discuss the multiple pro- and anti-inflammatory roles of B lineage cells play in autoimmune diseases, in the context of recent findings using B lineage targeting therapies.

## Introduction

The presence of autoantibodies is characteristic of most autoimmune diseases and has been widely used for diagnosis. Despite this, within the last 10–15 years B cells have been recognized as therapeutic targets for the treatment of autoimmune diseases. B cell subtypes, and the mechanisms of antibody production and maintenance, are highly diverse, and likewise the susceptibility of autoantibody-secreting plasma cells to therapies seems to be dependent on their tissue localization (1). Generally, conventional immunosuppressive therapy, using either steroids or cytostatic drugs, is commonly used in many autoimmune diseases and partly inhibits autoantibody production (2–5). At present, several drugs that specifically target B cells or plasma cells are either in clinical use or under development and promise to be very efficient for the treatment of various autoimmune diseases (6–8). Among them are (I) monoclonal antibodies against CD19, CD20, and CD22 that can directly target multiple B cell subtypes, but not or only to a lesser extent mature antibody-secreting plasma cells, (II) inhibitors of B cell activating factor (BAFF) and A proliferation-inducing ligand (APRIL), two cytokines which are very important survival factors for B cells and plasma cells, respectively, and (III) velcade/bortezomib, a small molecule proteasome inhibitor that spares B cells but eliminates both short-lived and long-lived plasma cells (9).

B cell directed therapies have proven not only to be therapeutically effective in classic B cell/autoantibody-driven disorders, such as autoimmune blistering skin diseases, myasthenia gravis, or antibody/immune-complex-mediated systemic lupus erythematosus (SLE), but also in diseases that are believed to be mainly driven by T cells, most prominently rheumatoid arthritis (RA) or multiple sclerosis (MS) (10–12). By contrast, in some cases, therapeutic B cell depletion results in the aggravation of symptoms. These findings emphasize that B cells play multiple roles that are relevant for the onset and clinical outcomes of autoimmune inflammatory disorders.

In this review, we will discuss how B cell targeting therapies may affect distinct B cell and plasma cell subpopulations, and how this depletion modulates the outcome of autoimmune diseases.

## B Cells, Plasma Cells, and their Impact on Autoimmune Diseases

### B Cell Maturation and Subsets

In humans and mice, there are three known functionally and phenotypically distinct B cell subsets: B-1, B-2, and marginal zone (MZ) B cells (13). While B-1 and MZ B cells can contribute to innate and adaptive immunity, “conventional” B-2 cells provide adaptive humoral immunity.

B-1 cells arise already early in embryonic development and comprised about 5% of all B cells in mice and humans. The B-1 population is the major source of natural IgM antibodies that exhibit reactivity to self and common microbial antigens (14). B-2 cells are activated in T-dependent immune responses and produce antibodies of all subclasses, and are capable of forming memory B cells with increased antibody affinity. MZ B cells are found in the marginal sinus of the white pulp of the spleen and predominantly produce antibodies that are specific for carbohydrate antigens.

While autoantibodies contribute to the pathogenesis of many autoimmune disorders, natural autoantibodies can be protective (14–16), suggesting that the various B cell subsets play multifaceted roles in autoimmune diseases (17).

After birth, large numbers of immature B-2 B cells are continuously formed within the bone marrow (18). During the stepwise differentiation of B cell precursors into immature B cells, the genes encoding the B cell receptor are reorganized, which at the population level generates a heavily diverse antibody repertoire. Immature B cells express high levels of functional antigen receptors (antibodies) of the IgM subclass on their surface (19). Thereafter, based on the specificity and affinity of their individual B cell receptors, immature B cell clones are either negatively or positively selected (20).

Some immature B cells bearing an autoreactive B cell receptor become deleted by central tolerance mechanisms operative in the bone marrow. The immigration of immature B cells into the spleen occurs through the terminal branches of the central arteriole that drains into sinusoids of the MZ (21). After migration to the splenic follicles, non-self-reactive IgM^+^/IgD^−^ immature B cells penetrate the MZ sinus to migrate through the interface between the periarteriolar lymphoid sheaths and B cell follicles, and eventually become IgM^+^/IgD^+^ naive mature B-2 or MZ B cells. This process is strongly dependent on high concentrations of the cytokine BAFF, which is expressed in splenic follicles. Most self-reactive immature B cells that passed central tolerance induction are energized or deleted in the spleen before they reach the BAFF-rich follicles. This peripheral tolerance mediating process relies on the activation of self-reactive B cells by autoantigens, which results in arrest of migration before these cells can enter the splenic follicle, and as a consequence they die as a result of BAFF-deprivation (21). Despite this, autoantibodies are also found in healthy individuals (22, 23), suggesting that some autoreactive B cells escape both the central and peripheral tolerance inducing mechanisms.

Mature B cells recirculate through the blood and accumulate in the follicles of secondary lymphoid tissues (24). Interestingly enough, mature B cells express a certain degree of migrational diversity. While all mature B cells are found in systemic and mucosal secondary lymphoid tissues, only a subpopulation of mature B cells recirculate through the bone marrow, a process of unknown biological significance, but which is clearly well controlled. In mice, these B cells express CD22, an Ig superfamily member serving as an adhesion receptor for sialic acid-bearing ligands. CD22 binds to CD22-ligands specifically present on sinusoidal endothelial cells in the bone marrow, but not on endothelial cells in other lymphoid tissues (25). A heterogeneous population of B cells also recirculates through the skin, as has been shown in animal models (26). These B cells express typical skin-homing receptors, e.g., ligands for E-selectin and α-4 and β-1 integrins, and exhibit a different immunophenotype than lymph node B cells. It is thought that these cells activate T cells at the site of inflammation and can increase local antibody production (26). However, the possibility that skin-homing B cells contribute to autoimmune skin diseases remains to be established.

After stimulation with their cognate (self-) antigen, mature B cells eventually may form short-lived plasma cells, memory B cells, or long-lived plasma cells (27, 28). As discussed below, these cell types markedly contribute to the pathogenesis of autoimmune diseases and exhibit a specific response to distinct therapeutic approaches.

### Memory B Cells

Memory B cells are formed within germinal centers and differ from naive B cells with respect to several features. While many human and murine memory B cells still express IgM, a significant proportion has already switched to the production of downstream antibody subclasses, mainly IgA and IgGs (29–31). Antigen-mediated crosslinking of membrane IgG provides a much stronger activation signal than IgM (32), which contributes to the reduced activation threshold observed for memory B cells and their capacity to quickly give rise to antibody-secreting plasma cells (33–36). Independent from their subclass, memory B cells have down-regulated the expression of genes that negatively control BCR signaling and have up-regulated the expression of their counterparts (37, 38). Moreover, memory B cells also express higher affinity B cell receptors, which not only strengthens the effector functions of the antibodies secreted by their plasma cell progeny, but also allows memory B cells to sense very low antigen doses. Consequently, memory B cells resemble very powerful antigen-presenting cells (APCs) (39, 40). While dendritic cells definitively represent the most important APC during the initiation of the immune response, memory B cells may take over later. Hence, antigen-presentation by memory B cells might be of particular importance during the later phases of chronic immune reactions, such as autoimmune diseases. Human, but not murine, memory B cells show increased expression of CD27. The interaction of CD27 with its ligand on T cells, CD70, promotes the differentiation of activated memory B cells into plasma cells (41). Compared with naive cells, human memory B cells also exhibit a distinct expression profile of homing molecules; these molecules help B cells to interact with T cells and present their cognate antigen in the context of MHC class II in an optimal manner (42–47). The capacity to efficiently interact with T cells is crucial to quickly boosting both the formation of plasma cells and antibody secretion, but likely also increases the capacity of memory B cells to modulate T cell responses.

In conclusion, memory B cells are optimized to interact with T cells and to yield strong antibody responses even in response to relatively little stimulation. Autoreactive memory antibodies are likely to contribute to the chronic and progressive course typically observed for autoimmune disorders (48). High frequencies of memory B cells are associated with poor clinical responses to RTX treatment (49). RTX treatment results in the efficient depletion of memory B cells in peripheral blood, and relapse after this treatment is thought to be associated with the repopulation of IgD-CD27^−^ and IgD-CD27^+^ memory B cells (50). However, RTX may not deplete the whole B cell memory compartment. RTX-treated patients can generate robust recall responses to repeated influenza vaccination, as indicated by the increase in serum antibody levels and peripheral blood plasmablast frequencies (51). This memory response is comparable to that observed in healthy controls. Hence, a significant fraction of memory B cells seems to be resistant to RTX treatment and is most likely localized in lymphoid or inflamed tissues (51). In mice, distinct layers of memory B cells have been identified, suggesting that the memory compartment is much more complex and diverse than expected (52, 53). Similarly, in human donors, distinct subpopulations of memory B cells that distinctly express homing receptors, such as CXCR3, have been observed in blood (46, 54). In response to IFN-γ, activated B cells upregulate CXCR3, which thereafter is stably expressed on their memory B cell progeny (46, 54). These findings may indicate that a subpopulation of memory B cells is formed under inflammatory conditions, such as during an autoimmune disease flare-up, which then might be able to migrate into inflammatory tissues where they are relatively protected from therapeutic intervention.

### Short-Lived and Long-Lived Plasma Cells

Successful activation of naive B cells leads to a massive clonal expansion and eventually the formation of short-lived plasma cells in the extra-follicular areas of secondary lymphoid tissues (55). In mice, these cells exhibit lifetimes of less than a week (56). Cytostatic drugs, such as cyclophosphamide, prevent B proliferation and the formation of new antibody-secreting cells, hence eliminating the short-lived plasma cell compartment (57). Simultaneous to the formation of short-lived plasma cells, germinal centers may give rise to both memory B cells and long-lived plasma cells. For a brief period following vaccination, precursors of long-lived plasma cells (plasmablasts) are found in human peripheral blood (54); these cells then give home to deposit tissues, such as bone marrow, mucosal tissue, or sites of inflammation. The tissue homing of these cells is tightly regulated by adhesion molecules, chemokines, and their receptors (58–60). In peripheral blood, plasma cell subpopulations are found that exhibit different expression profiles of homing receptors. For example, migratory plasmablasts, induced by intracutaneous vaccination, express L-selectin, which are associated with homing to peripheral lymph nodes (61, 62). Similar to the return of locally activated plasma cell precursors to mucosal sites, cells that are activated in the context of a pro-inflammatory response seem to be programmed to relocate to inflamed tissues. Here, the high levels of inflammatory cytokines can support plasma cell survival, likely enabling these cells survive for the period of inflammation (60). These data suggest that plasma cells formed in the course of an inflammation-associated immune response can maintain the production of antibodies but will disappear when the cause of inflammation has resolved (46, 63).

In human tissues, various plasma cell subpopulations exist which exhibit different phenotypes and stages of differentiation (46, 64, 65). Some plasma cell stages still express CD20, indicating that they might be susceptible to RTX treatment. However, mature human plasma cells have lost CD20 expression (66–68). Accordingly, even B cell depletion has been found to have no impact on the production of human memory antibodies, e.g., specific for tetanus toxoid (63). In general, serum antibodies of different specificities show a great variety of responses following B cell depletion, ranging from no response to depletion below the detection limit (69), possibly suggesting that antibodies are maintained by various mechanisms, including short-lived plasma cell populations that are replenished by various memory B cell subsets and by long-lived plasma cells. The notion that long-lived plasma cells contribute to the production of autoantibodies (57), but are not affected by conventional immunosuppressive drugs such as steroids or cyclophosphamide, or by B cell depletion, has identified them as a novel target cell requiring specific therapeutic approaches (70).

### B Lineage Cells Exert Multiple Roles That Drive Pathogenesis but Also Control the Severity of Inflammation

B cells play multiple functions. Following differentiation into plasma cells, they secrete huge amounts of antibodies into the body fluids. Autoantibodies can contribute to the pathogenesis of autoimmune diseases in multiple ways (71). They can initiate immune-complex-mediated inflammation, deplete specific cell types or modulate important signaling pathways, relevant in SLE, antibody-mediated hemolytic anemia or Hashimoto’s thyroiditis and Graves’ disease, respectively (72–75). However, more recent studies indicate that antibodies can also exert significant anti-inflammatory effects, which limit or even inhibit autoimmune pathogenesis. The pro- and anti-inflammatory effects of antibodies depend on their isotype (76) and on their Fc *N*-linked glycosylation patterns (77, 78). While IgGs with low levels of galactosylation promote inflammation, sialylated IgGs have a strong anti-inflammatory capacity (79, 80). Highly glycosylated IgG antibodies have been shown to inhibit autoimmune inflammation in mouse models (76, 81, 82). Changes in autoantibody glycosylation have been observed during the course of human autoimmune diseases, possibly providing an interesting novel diagnostic tool, but also suggesting that the antibody glycosylation pattern can alter the clinical course of autoimmune disorders (83).

B lineage cells can also present antigens in the context of MHC II and secrete immunomodulatory cytokines, thereby playing a prominent role for the modulation of antigen-specific T cell responses (84–86). B cells probably only play a minor role in T cell priming. However, during secondary or chronic immune reactions B cells resemble very potent APCs that drive the expansion of activated T helper cells (87–90). Hence, B cells are likely to be both important antigen-presenting and T cell promoting cells during chronic and repeated immune reactions, such as occurs during the course of autoimmune diseases.

In addition, various B lineage cells, including those with a CD138^+^ plasmablast/plasma cell phenotype, have been shown to express a variety of pro-inflammatory cytokines that can stimulate innate effector cells and significantly contribute to inflammation and immune protection in murine models (91–93). Moreover, while some B lineage cells promote inflammation, others exhibit profound immunosuppressive capacities (94). As shown in many models, B cells and plasma cells can also suppress autoimmune inflammation through the production of cytokines, such as IL-10, TGF-beta, or IL-35 (86, 95–98). Interestingly, IL-10 and IL-35 are produced by B cells that have different phenotypes (98). As discussed below, the therapeutic induction of immunosuppressive B lineage cells may be an interesting direction for the development of future therapies.

## Lessons from RTX Treatment of Autoimmune Diseases

Regarding the clinical use of B cell targeting therapies, the majority of the information comes from studies using RTX. This chimeric mouse/human monoclonal antibody targets the pan B cell marker CD20, a transmembrane protein expressed on all B lineage cells, from early pre-B to mature B and memory B cells. CD20 has been shown to mediate Ca^2+^ influx across plasma membranes and is important in maintaining intracellular Ca^2+^ concentration and activation of B cells (99). RTX was the first anti-CD20 antibody approved by the U.S. Food and Drug Administration for medical use in 1997 as Rituxan^®^, originally to treat B cell non-Hodgkin lymphomas (100). Later it was also approved for use in RA, granulomatosis with polyangiitis, and microscopic polyangiitis and there is growing clinical use of RTX in other autoimmune diseases, such as MS, SLE, and autoimmune blistering skin diseases (101–105).

### Mode of Action of RTX

After binding of RTX to membrane bound CD20 it mediates strong complement-dependent cytotoxicity directed to its target cell due to the enhanced clustering of antibody Fc regions (106, 107). Based on its ability to redistribute CD20 into lipid rafts, which provides the molecular basis for RTXs engagement of complement factors, RTX is classified as a type I anti-CD20 antibody. By contrast, type II antibodies cannot cause this redistribution of CD20 (108–111), do not induce complement-dependent cytotoxicity to the same extent (111), but appear to induce a greater degree of directly induced, non-apoptotic cell death, upon binding to target cells (112).

CD20 expression is lost during differentiation into mature antibody-secreting plasma cells (66–68). This lack of CD20, particularly on long-lived plasma cells, explains why RTX treatment does not interfere with the production of memory antibodies, such as anti-tetanus/-measles/-mumps/-rubella (63, 113). Depending on the stage of plasma cell differentiation and tissue localization, early plasma cells (plasmablasts) exhibit various levels of CD20 expression (114). While mature long-lived plasma cells are apparently not depleted by RTX, it is not known to which extent earlier plasma cell stages are affected.

Independent of disease, RTX leads to the depletion of peripheral B cells from approximately 90% to close to 100% (Table 1). Despite this fact, the clinical efficacy of RTX varies broadly among different autoimmune diseases, and also among individual patients. Following withdrawal of RTX treatment, B cell levels recover within 6–20 months, with the rate of recovery greatly varying between individual patients (115).

**Table 1:** Efficacy of RTX treatment varies.

		RA		SLE		PV	MS		
Reference		Emery et al. (11); Rubbert-Roth et al. (105); Haraoui et al. (119)	Rovin et al. (145)	Leandro et al. (146); Albert et al. (147)	Lu et al. (148)	Ahmed et al. (116); Joly et al. (103); Pfütze et al. (117)	Dunn et al. (150); Cross et al. (104)	Hauser et al. (149)	Hawker et al. (151)

Patient no.		120/346/465	144	24	50	11/21/11	16/399	104	439

Dose and duration		1 mg for 24/48 weeks	1 mg for 52 weeks	1 mg for 6 months	1 mg for 6 months	375 mg/m^2^ for 6 months	375 mg/m^2^/0.5–1 mg for 6 months	1 mg for 48 weeks	1 mg for 96 weeks

Clinical improvement	Partial Complete No	*ACR20/50/70*: in 54–72/27–48/7–23% – in 5%	*Proteinurea*: in 26.4% in 30.6% in 43.1%	*Proteinurea/BILAG/SLEDAI*: in 65–70% – –	*BILAG*: in 42% – –	*Skin lesions*: in 8.2–20% in 80–95% –	*EDSS*: in 12.5–63.2% – in 36.8–81.25%: worse in 6.25%	*GELN*: in 80.3% in 19.7% –	*CDP*: – – in 100%

B cell depletion efficacy in periphery	Significant depletion to 6 cells/μL	Complete depletion in 99%	Almost complete depletion in 94–96% of patients	Complete depletion in 42%; partial depletion in 47% and no depletion in 11% of patients	In almost all patients complete B cell depletion	Depletion by 95–99.8% in all patients; 90% depletion in spinal fluid	Over 95% depletion in all patients	

Autoantibody involvement	Anti-CCP aab and RF reduced by 45%	Anti-dsDNA aab reduced by 75%	Anti-dsDNA aab reduced by 35%	Anti-dsDNA aab reduced by 60%	In 81.8% IgG/IgG4 anti-keratinocyte cell-surface aab undetectable; dramatic decrease of IgG/IgG4 anti-Dsg1 (by 80%) and Dsg3 (by 65%) aab	Elevated serum anti-MOG aab	–	

Remark		HACA in 2.3–7.3%	–	HACA in 33%, correlating with B cell depletion rate		Clear correlation between aab and disease	HACA in 24.1–37%, correlates with B cell depletion rate	HACA in 24.1% of patients	

*RA, rheumatoid arthritis; SLE, systemic lupus erythematosus; PV, *Pemphigus vulgaris*; MS, multiple sclerosis; ACR, American College of Rheumatology Criteria, standard criteria to measure the effectiveness of arthritis treatments in clinical trials for rheumatoid arthritis, 20/50/70 refers to the improvement in tender or swollen joint counts as a percentage; HACA, human anti-chimeric antibodies; CCP, cyclic citrullinated peptides; RF, rheumatoid factor; MTX, methotrexate; MMF, methyl mofetil; Std, standard therapy; BILAG, British Isles Lupus Activity Group, organ-specific 86-question assessment based on the principle of the clinical intent to treat; SLEDAI, SLE Disease Activity Index, a list of 24 items (16 clinical items including seizure, psychosis, organic brain syndrome, visual disturbance, other neurological problems, hair loss, new rash, muscle weakness, arthritis, blood vessel inflammation, mouth sores, chest pain that worsens with deep breathing, and manifestations of pleurisy and/or pericarditis and fever, and eight laboratory results, including urinalysis testing, blood complement levels, increased anti-DNA antibody levels, low platelet count, and low white blood cell count); EDSS, Expanded Disability Status Scale is a method of quantifying disability in multiple sclerosis and monitoring changes in the level of disability; CDP, delayed time to confirmed disease progression; Dsg, desmoglein; cANCA, anti-neutrophil cytoplasmic antibodies; MOG, myelin oligodendrocyte glycoprotein; aab, autoantibodies; dsDNA, double-stranded DNA*.

### Use of RTX in Pemphigus Vulgaris (PV)

RTX has shown promising results in the treatment of PV, an autoantibody-driven blistering skin disease. In three independent clinical studies, comprising a total of 43 patients with this rare autoimmune disorder, RTX treatment achieved a complete remission in over 80–95% of patients (103, 116, 117) who, importantly, were refractory to steroid therapy. This impressive clinical outcome was achieved in parallel with B cell depletion close to undetectable levels in almost all patients. One study showed that in most, but not all patients, the levels of anti-keratinocyte cell-surface IgG4 autoantibodies dropped to undetectable levels (116). In another study, anti-desmoglein 1 and 3 (Dsg1/3) autoantibodies were measured and found to be reduced on average by 65–80% (103). Generally, the response to RTX treatment seems to correlate with the extent of B cell depletion. However, two treated patients showed clinical remission despite persistent high levels of anti-Dsg1/3 autoantibodies, although remission was delayed compared with that in patients who showed remarkably reduced autoantibody levels (103). Hence, a reduction in the levels of autoantibodies seems to be a major factor in the success of RTX in treating PV. Nevertheless, the finding that RTX can improve clinical symptoms in patients that still exhibit high levels of anti-Dsg1/3 autoantibodies, suggests that RTX can also act *vi*a additional mechanisms. It would be interesting to consider this concept while designing future clinical studies.

### Use of RTX in RA

Studies using RTX in RA have provided additional evidence that its therapeutic efficacy is not merely based on the reduction of autoantibody levels. In this disease, RTX is recommended for use in patients refractory to standard therapy (118). Although the B cell depletion rate is almost 100%, a clinical response was observed in approximately 60–70% of patients (11, 105, 119). Considering that these patients did not respond to other therapies, this was deemed to be a significant success. Results from more recent studies have suggested that RTX has better long-term efficacy when used in patients with fewer previous treatments and lower disease activity (120). Interestingly, the clinical response to RTX in RA positively correlates with the presence of anti-CCP autoantibodies, but is inversely correlated with the IgG-levels present before treatment (121–124). Autoantibodies and IgG-levels together with serum IL-33 have also been reported to predict the clinical response to RTX (125).

Based on the correlation between serum autoantibodies and response to RTX, on first view it seems possible that suppression of autoantibodies is a relevant mechanism by which RTX affects the clinical outcome of RA. The presence of autoantibodies such as rheumatoid factor (RF) and anti-CCP correlate well with disease activity in RA patients and indicate the presence and activation of autoreactive B cells. Moreover, anti-CCP and IgM-RF can enhance pro-inflammatory macrophage functions *in vitro* (126), supporting the idea that these autoantibodies may also contribute to RA pathogenesis *in vivo*. However, the reduction of serum autoantibody levels in RTX-treated responders is only very moderate, i.e., approximately 50 and 30%, for anti-CCP and RF, respectively, with a high degree of variation being observed (69). It seems questionable that such minor reductions in autoantibody levels cause the clinical response. Moreover, the absolute autoantibody levels persisting in responders after treatment were still two to fivefold higher than in non-responders (69). If autoantibodies in responders and non-responders exhibit similar pathogenicity, the moderate depletion of autoantibodies in responders to levels above of non-responders would not explain the success of the therapy. Other studies have also shown very moderate RTX effects on IgA and IgG anti-CCP autoantibodies, suggesting that a significant proportion is produced by long-lived plasma cells (127). The question of whether these antibodies contribute to RA pathogenicity remains to be addressed.

### RTX Effects on CD4 T Cells

Alternative explanations for the anti-inflammatory effects of RTX in RA patients include the suppression of inflammatory T cells in favor of regulatory T cells subsets, possibly in combination with an induction of high galactosylated anti-inflammatory antibodies. There is good evidence that CD4 T cells can activate myeloid and synovial cells, which in turn activate and recruit macrophages to synovial tissue, eventually leading to joint inflammation and cartilage destruction, whereas regulatory T cells are protective (128–131).

The production of pro- and anti-inflammatory cytokines such IFN-γ, IL-17, or IL-10 seems to be key for T cell mediated control of RA pathogenesis. There is evidence that the clinical response to RTX therapy in RA is associated with lower IFN-γ levels (132). Moreover, a study including 52 patients showed that RTX often induces a reduction in the number of peripheral blood CD4 T cells, an effect that was strongly associated with the clinical response (133). It is possible that the depletion of a small subpopulation of CD20^+^ T cells contributes to this effect (134).

However, there is good evidence that B cells can efficiently promote CD4 T cell functions through antigen-presentation and cytokine release. As discussed above, while B cells may play only a minor role in T cell priming, during secondary or chronic immune reactions, B cells can act as very potent APCs that drive the expansion of activated T helper cells (87–89, 135). Hence, B cell depletion may impair the formation, clonal expansion, and function of T memory cells.

Accordingly, T cell activation in the synovium of RA patients has been reported to be dependent on B cells (136). Hence, it is highly likely that RTX mediates its beneficial effects in RA at least partly through the depletion of T cell stimulating B lineage cells, although this remains to be studied further.

### Effects of RTX Mediated B Cell Depletion on Cytokines

Of note, under certain conditions, B cells can also directly promote innate inflammatory effector cells through the production of cytokines such as IFN-γ, IL-6, granulocyte-macrophage colony-stimulating factor, and IL-17 (91, 93, 137, 138). In mice, B cell ablation has been shown to ameliorate autoimmune diseases by depleting IL-6-producing B cells (93). Moreover, a recent study has provided evidence that B cells from RA patients show abnormal IL-6 signaling and altered cytokine production, and that this may contribute to disease (139). Hence, B cells resemble an underestimated source of inflammatory cytokines and the depletion of such pro-inflammatory cytokine producing B cells is likely to contribute to the therapeutic outcome of RTX treatment.

### RTX Effects on Antibody Glycosylation

T cell help in germinal centers has been found to be important for the induction of inflammatory low-glycosylated IgG antibodies (82). In particular, the T cell-derived cytokines such as IFN-γ and IL-17 are capable of synergistically promoting the production of low-sialylated, and potentially pathogenic, antibodies (82, 139). Based on the notion that the clinical response to RTX in RA is associated with lower IFN-γ levels (132), it is possible that a reduction in IFN-γ levels, in turn, leads to alterations in antibody glycosylation.

In accordance with the idea that these processes are of pathophysiological relevance, reduced antibody glycosylation has been reported to precede disease onset and to correlate with disease activity in RA patients (140). This phenotype is highly prevalent in, but not restricted to, autoantibodies (141). Hence, it is possible that acting through a reduction in IFN-γ levels, RTX corrects the shift toward the production of pro-inflammatory antibodies that is observed in RA. Of note, high-glycosylated antibodies can mediate anti-inflammatory effects independent of their specificity, as indicated by the therapeutic effects of intravenously administered immunoglobulins (142), although antigen-specific effects might be considerably stronger and require lower antibody concentrations (79, 81, 143). Accordingly, highly glycosylated IgGs have been reported to induce antigen-specific tolerance (78). Hence, RTX may partly mediate its beneficial effects through changes in antibody glycosylation, either *via* a reduction in autoantibody pathogenicity, through the generation of anti-inflammatory autoantibodies, or by the induction of persistent high levels of anti-inflammatory total IgGs that mimic continuous IVIG treatment.

### Effects of RTX in MS and SLE

Similar to what has been observed in RA, the therapeutic effect of RTX in SLE and MS is variable. Its impact on total antibody levels as well as on autoantibody levels shows a high degree of diversity (Table 1). In a recent study, only 11 out of 32 SLE patients with IgG hypergammaglobulinemia before treatment showed reduced IgG-levels after 12 months of treatment (144). Likewise, a reduction in anti-double-stranded DNA levels was incomplete, with high inter-individual variety and differences between antibody subclasses (145–148). Despite homogenous B cell depletion rates in MS of over 90 and 95% in spinal fluid and in the periphery, respectively, the disease outcome showed great variation (104,  149–151). Interestingly, RTX has even been found to worsen the clinical outcome of MS (104).

These variable results might be not be surprising in the light of the finding that B lineage cells play multiple pro-and anti-inflammatory roles in experimental autoimmune encephalomyelitis (EAE), a murine model of MS. B cell-derived IL-6 has been shown to be crucial for the initiation of EAE, suggesting that B cells can promote MS pathogenesis through the production of this pro-inflammatory cytokine (93). However, there is an abundance of evidence that anti-inflammatory B cell subsets can also efficiently suppress CD4 T cells mediating neuroinflammation, and that these effects are mediated by B lineage-derived IL-10, TGF-β, and IL-35 (98, 152). These findings led to the concept of regulatory B cells (Bregs), which, however, have never been clearly defined. Recent results indicate that these IL-10^+^ B lineage cells have a plasmablast phenotype (98, 153). Similarly, investigations conducted by our group have identified plasmablasts/plasma cells as an important source of IL-10, capable of suppressing skin inflammation in a murine model of epidermolysis bullosa acquisita (EBA) (85). In EAE, B lineage-derived IL-6 and IL-10 were shown to have an impact on the induction and resolution of inflammation, respectively (93, 98, 153). These findings may partly explain the heterogeneity of the clinical response to RTX observed in MS. Depending on the major role of B lineage cells as drivers or inhibitors of inflammation in individual patients, and possibly related to timing, RTX may be either beneficial or worse for the clinical course of MS.

## Alternative B Cell Targeting Approaches

### Second Generation Anti-CD20 Antibodies

The great clinical success of the chimeric antibody, RTX, has stimulated the development of the second generation anti-CD20 antibodies, ocrelizumab, obinutuzumab, veltuzumab, and ofatumumab (154). These second generation anti-CD20 antibodies are humanized or even fully human, exhibit improved effector functions, and compared with rituximab show greater potential *in vitro*. Due to these properties, they are expected to be more effective, to exhibit lower immunogenicity, and to be better tolerated. However, these expectations, which may be validated by head-to-head trials of these second generation anti-CD20 antibodies and RTX, have not been confirmed till date.

Ocrelizumab has recently been approved for the treatment of relapsing-remitting MS, and is the first approved treatment for primary progressive MS. In RA, however, ocrelizumab seems to have no benefit over current treatments, leading to a halt in the development of ocrelizumab for the treatment of RA (155).

Clinical trials exploring the use of obinutuzumab, veltuzumab, and ofatumumab in autoimmune disorders are at various stages of development and have been extensively reviewed by Du and colleagues (154). In general, it appears that the new anti-CD20 antibodies are effective against autoimmune diseases, against which RTX is also beneficial, but more studies are needed to evaluate their efficiency and long-term safety profiles in greater detail.

### Antibodies Targeting CD19

Other B cell depleting reagents that promise a great potential for the treatment of autoimmune diseases are a series of reagents targeting CD19, which are currently under development. These include humanized antibodies, “bi-specific T cell engagers,” and antibody-drug conjugates, such as inebilizumab, blinatumomab, and SAR3419, among several others (156, 157).

CD19 targeting therapeutics generally exhibit a broader target spectrum than anti-CD20 based reagents. CD19 is expressed very early in B cell development, being evident already on pro-B cells and on all later B cell stages, whereas CD20 is expressed later, starting at the immature B cell stage. Possibly of greater importance for the treatment of autoimmune diseases, in addition to all mature B cell subsets, CD19 is expressed on a significant proportion of plasmablasts and plasma cells, particularly outside the bone marrow (64, 114). Accordingly, CD19 targeting reagents have been reported to deplete pre-existing peripheral antibody-secreting cells, at least in humanized mouse models (158). Hence, CD19 targeting reagents might be not only suitable for treating B cell malignancies, but also exhibit great potential for therapeutic use in autoimmune diseases, particularly for those diseases with a strong involvement of pathogenic antibodies, e.g., SLE, pemphigus, and neuromyelitis optica.

### BAFF/APRIL Antagonists

An alternative therapeutic strategy to target B cells is the blockade of B lineage survival factors, such as BAFF and its homolog APRIL and their receptors (159). Together, BAFF and APRIL along with their receptors form a complex system, which is very important for the survival of mature B cells and plasma cells. There are three receptors that bind BAFF and APRIL with different affinities. BAFF binds to BAFF-R, transmembrane activator and calcium modulator and cyclophilin ligand interactor, and B cell maturation protein. These three receptors are differentially expressed at various times during B cell ontogeny (160). Most BAFF circulates as a soluble active homo-trimer (161) that binds to BAFF-R and this interaction is required for survival of late transitional, MZ, and mature naive B cells, all of which are depleted by BAFF-blockade (162, 163).

Several BAFF/APRIL targeting drugs are currently under development. The humanized anti-BAFF antibody belimumab has already been approved in the EU and the USA for the treatment of adult patients with active, autoantibody-positive, and SLE despite standard therapy. It is generally well tolerated with low rates of immunogenicity. Belimumab in combination with standard therapy reduces the overall disease activity and the incidence and severity of flares, has steroid-sparing effects, and can maintain disease control for at least 10 years (164). Interestingly, despite its clinical efficiency, belimumab only partly inhibits the production of IgG-autoantibodies. While some studies found no reduction of IgG-autoantibody levels following treatment with belimumab, others have reported a decline of 40–60% within 2–7 years of treatment (165–167). This is in accordance with the finding that belimumab depletes both naive and activated B cells, but not memory B cells (165, 167).

Similar to what has been observed with RTX, B cell targeting by blockade of BAFF and APRIL using belimumab, tabalumab, or atacicept also shows greatly variable effects on the levels of autoantibodies and the clinical outcome of autoimmune diseases (118, 168–181), and of note, seem to have only limited effects on the production of (auto)antibodies (Table 2).

**Table 2:** Efficacy of belimumab, tabalumab, and atacicept.

		RA			SLE				SS	MS
					
		Atacicept	Belimumab	Tabalumab	Atacicept	Belimumab	Blisibimod	Tabalumab	Belimumab	Atacicept
Patient no.		311	415	1,041	47/6	1,353	547	1,124	30	255

Duration		38 weeks	24/48 weeks	52 weeks	9/52 weeks	52–76 weeks	24 weeks	52 weeks	52 weeks	36 weeks

Clinical improvement	Partial Complete No	– – *ACR20*	*ACR20*: in 41% – *ACR50+70*	*ACR20/50/70*: in 70/36/13% – –	in 22.2% in 44.5% worse in 33% stopped	*SELENA–SLEDAI/BILAG*: in 46.5/58.6% – –	*Proteinurea*: reduced – –	*SRI-4*: in 49.2% – secondary end point	*EULAR*: in 86.7% – –	– – Failed and even worse

B cell depletion efficacy in periphery		Circulating mature B and plasma cells reduced	B cell depletion 16–48%; no depletion of memory B cells and plasma cells	B cell reduction by 18–40%; no depletion of memory B cells	Reduction by 60%; plasma cells depleted	Reduction by 55.7%	Significant reduction	Significant reduction	Significant reduction	Significant reduction

Autoantibody involvement		RF but not anti-CCP levels reduced	Reduction of RF by 30%	CRP reduced		Reduction of anti-dsDNA aab by 44–49%	Anti-dsDNA decreasedC3 increased	Anti-dsDNA aab significantly decreased	Reduction of RF by 30%	

Remark		Serum IgA+M (by 19.4%) and IgG (by 8.6%) modestly reduced	Moderate change of total Ig; better response in RF+ or ACPA+ patients	Total serum Ig decline by 11%Phase III study in RA terminated		Seropositive and highly diseased patients respond better; total serum Ig modestly reduced by 16%		C3 + C4 increased; total serum Ig reduced; development was stopped	Total Ig not changed	Severe adverse events; higher relapse rate in treated group compared to controls

References		Genovese et al. (170);van Vollenhoven et al. (171)	Stohl et al. (181)	Smolen et al. (168);Greenwald et al. (169)	Dall’Era et al. (176); Lenert et al. (177); Ginzler et al. (178)	Navarra et al. (172); Furie et al. (173)	Furie et al. (175)	Merrill et al. (174)	Mariette et al. (179)	Kappos et al. (180)

*RA, rheumatoid arthritis; SLE, systemic lupus erythematosus; SS, Sjörgen’s syndrome; MS, multiple sclerosis; ACR, American College of Rheumatology Criteria, standard criteria to measure the effectiveness of arthritis treatments in clinical trials for rheumatoid arthritis, 20/50/70 refers to the improvement in tender or swollen joint counts as a percentage; CCP, cyclic citrullinated peptides; RF, rheumatoid factor; MMF, methyl mofetil; std, standard therapy; BILAG, British Isles Lupus Activity Group, organ-specific 86-question assessment based on the principle of the clinical intent to treat; SELENA, Safety of Estrogens in Systemic Lupus Erythematosus National Assessment; SLEDAI, SLE Disease Activity Index, a list of 24 items (16 clinical items, including seizure, psychosis, organic brain syndrome, visual disturbance, other neurological problems, hair loss, new rash, muscle weakness, arthritis, blood vessel inflammation, mouth sores, chest pain that worsens with deep breathing, and manifestations of pleurisy and/or pericarditis and fever, and eight laboratory results, including urinalysis testing, blood complement levels, increased anti-DNA antibody levels, low platelet count, and low white blood cell count); EDSS, Expanded Disability Status Scale is a method of quantifying disability in multiple sclerosis and monitoring changes in the level of disability; aab, autoantibodies; C3 + 4, Complement factors 3 and 4; ACPA, Anti-citrullinated peptide/protein antibodies; CRP, C-reactive protein; SRI-4, Systemic Lupus Erythematosus Responder Index for 4-point reduction due to SLEDAI; EULAR SS, The European League Against Rheumatism for Sjörgen’s Syndrome; Ig, immunoglobulins; dsDNA, double-stranded DNA*.

Generally, B cell targeting represents a powerful strategy for the treatment of autoimmune diseases. Its mechanism of action seems to be diverse and complex, and needs further elucidation.

## Direct Targeting of Plasma Cells by Bortezomib

As originally shown by us and others, in mice, memory antibodies are secreted by long-lived plasma cells (182, 183). The notion that these cells can contribute to the production of autoantibodies but do not respond to current therapeutic approaches (57), has led to the search for novel plasma cell targeting agents.

The small molecule proteasome inhibitor bortezomib promotes plasma cell apoptosis and is approved for the treatment of multiple myeloma. Nearly a decade ago, Voll and colleagues reported that this drug could also be useful for the treatment of antibody-mediated autoimmune diseases. They demonstrated that bortezomib efficiently depletes both short-lived and long-lived plasma cells and protects mice with lupus-like disease from nephritis (70). Its efficacy was later proven in various models of antibody-mediated autoimmune diseases (184–186). There is now increasing evidence that bortezomib can also efficiently deplete autoantibodies in patients, resulting in the improvement of clinical symptoms, as has been described for refractory primary Sjögren’s syndrome, refractory SLE, thrombotic thrombocytopenic purpura, and among others (9, 187–197). The potential development of severe side effects, such as peripheral neuropathies may limit the use of bortezomib in autoimmune diseases. However, its unique capacity to deplete antibody-producing plasma cells suggests that the safety and efficacy of bortezomib should be evaluated in clinical trials including more patients who are refractory to standard therapeutic approaches.

## Induction of Anti-Inflammatory B Lineage Cells: A Promising Therapeutic Treatment Option?

IL-10^+^ B lineage cells have been known as potent suppressors of autoimmune inflammation for decades (198). Over the last decade, the expansion of IL-10^+^ B cells using various approaches has been shown to efficiently suppress both autoimmune and allergic inflammation in numerous models (85, 199, 200). The first report that IL-10^+^ B cells exert a suppressive function was in 2002 by Fillatreau and colleagues (86), who showed that chimeric mice with a B cell specific IL-10 deficiency do not recover from EAE. Later, IL-10^+^ B cells were termed as Bregs or B10 cells. However, no surface marker or transcription factor unique to these cells has been identified to date. These cells are only functionally defined by their production of anti-inflammatory cytokines such as IL-10, and more recently IL-35, and the resulting suppression of inflammation and autoimmune diseases (201, 202).

### Phenotype and Origin of Human IL-10-Producing B Cells

The combination of markers used to describe “regulatory B cells” in human and mice is controversial. The phenotypic identification of these B cells and their possible origin and development have been excellently reviewed elsewhere (198, 203). In humans, the ability to produce anti-inflammatory IL-10 has been reported in B cells at various stages of development: immature/transitional B cells (CD19^+^ CD38^hi^ CD24^hi^) (204), plasmablasts (CD27^int^ CD38^hi^) (153, 205), and memory B cells (CD19^+^ CD27^+^) (206,  207). It is likely that IL-10^+^ B cells represent a transient stage with a functional program rather than a terminally differentiated stage, and that any B cell can acquire suppressive properties within a certain environment. Nevertheless, it is debatable if these cells arise from a single shared progenitor, from individual progenitors, or are induced under certain environmental stimuli (198, 203). Interestingly, in this context autocrine IL-10 can promote human IL-10^+^ B cells to differentiate into IgG- and IgM-secreting plasma cells (208).

### IL-10-Producing B Cells in Patients Suffering from Autoimmune Diseases

The first report describing human IL-10-producing B lineage cells in autoimmune diseases was in 2010 by Iwata et al. (207), who described abnormally high frequencies of peripheral IL-10^+^ B cells in various autoimmune diseases, such as SLE, RA, MS, Sjögren’s syndrome, and blistering skin diseases. An increase in the number of blood IL-10^+^ CD19^+^ CD24^hi^ CD38^hi^ cells was also found in PV patients, but these B cells were functionally unable to suppress Th1 immune responses (209). By contrast, several studies on RA (206, 210–212), SLE (204), systemic sclerosis (SSc) (213, 214), and MS (215–217) patients have shown a reduced number of peripheral IL-10-producing B cells compared with that in the controls. This was often accompanied by an impaired suppressive capacity of CD4 T cells. An overview describing the modulation of human IL-10-producing B lineage cells in different autoimmune diseases has been provided by Miyagaki et al. (218).

### IL-10-Producing B Cell Dynamics Following B Cell Targeting Therapy

In myasthenia gravis, depletion of B cells with RTX showed that IL-10^+^ B cells can be found to have repopulated in the periphery after several months (219). Immunosuppressive treatments, TNF-therapy, and BAFF-blockade in RA (206), SSc (213), and experimental diabetes mellitus type 1 (220), respectively, have shown that IL-10-producing B lineage cells enrich after treatment and that their frequency is even higher than before treatment. In relapsing-remitting MS, the frequencies of IL-10^+^ CD19^+^ B cells were significantly reduced in patients experiencing a relapse compared with that in patients in remission (217), indicating that the clinical outcome of the disease also depends on the availability of IL-10-producing B cells.

Moreover, a “good responder” to RTX in myasthenia gravis showed a rapid repopulation of CD19^+^ IL-10^+^ B cells after from 8 to 9 months compared with a “non-responder,” where repopulation was delayed (219). This shows that the kinetics of the IL-10^+^ B cell repopulation is related to the responsiveness to RTX. Similarly, Colliou et al. (221) have shown that RTX-treated PV patients in complete remission had fourfold higher numbers of IL-10^+^ CD19^+^ B cells compared with patients in incomplete remission. In SLE patients responding well to RTX treatment, IL-10^+^ CD24^hi^ CD38^hi^ B cells were found to repopulate and exhibited a restored suppressive function compared to non-responders (222).

Differences in IL-10-producing B cells in individual patients and types of autoimmune diseases could explain the differential outcome and benefit of B cell specific therapies. For example, in SLE and lupus nephritis, pan B cell therapies, such as RTX, show only moderate or even no benefit, despite the significant role of B cells in this disease (145, 223). The depletion of anti-inflammatory B cells could contribute to this unexpected result. Very few studies to date have included an analysis of the kinetics and function of IL-10-producing B cells after B cell depleting therapy. To better understand the individual clinical outcome of the patients and the differences between certain autoimmune diseases treated with the same B cell targeting agent, it would be of great benefit to include an analysis of IL-10^+^ B lineage cells in further studies.

### Challenges Hampering the Development of IL-10^+^ B Cell-Based Therapies

Restoring the regulatory capacity and the number of IL-10^+^ B cells is a promising therapeutic goal for the treatment of autoimmune diseases. However, currently two unsolved problems hamper the development of a therapy based on IL-10^+^ B cells. First, the methods used to generate IL-10^+^ B cells for therapeutic approaches are not suitable for a clinical setting. Second, the identity and phenotype of IL-10^+^ B cells remain uncertain. Nevertheless, recent progress has been made with respect to both issues. Giacomini et al. (224) have shown that stimulation of peripheral blood mononuclear cells (PBMCs) from MS patients with thymosin-α1 (Tα1) increases IL-10 and IL-35 secretion and expands transitional- and plasmablast-like B cell populations. Upon exposure to pro-inflammatory cytokines, such as IL-21, IL-6, and IL-1β, an expansion of IL-10^+^ B cell population has been observed. In RA patients, IL-21 increases the number of IL-10-producing B cells in the memory compartment and induces IL-10^+^ plasmablasts (206), whereas in mice, gut microbiota-derived IL-1β and IL-6 promote the formation of various IL-10^+^ B lineage cells in the spleen and lymph nodes (225). By contrast, the anti-inflammatory cytokine IL-35 can also induce human B cells to produce IL-10 and IL-35 (226). Nevertheless, inducing anti-inflammatory B cells *in vivo via* inflammatory cytokines bears the risk of undesirable pathogenic side effects by also activating other effector cell types. If not expanded *in vivo*, IL-10^+^ B cells could be also induced from patient PBMCs *in vitro* and transferred back. Here, the questions of the amount of B cells required to improve clinical symptoms and the stability of the IL-10^+^ phenotype and function arise. The difficulties and potential of these therapies were recently discussed by Mauri and Menon (227).

### Induction of IL-10-Producing Plasma Cells/Plasmablasts: Potential as a Novel Treatment Option

Progress has been made in defining the identity of IL-10^+^ B cells that could be used to develop a novel therapeutic strategy. During the last decade, several phenotypically distinct murine B cell subsets have been described that produce IL-10 upon *in vitro* stimulation, which was able to limit autoimmune diseases (198). These cells include B cells with a CD5^+^ CD1d^hi^ phenotype (B10) (228), CD5^+^ B cells (B1-a) (229), transitional type 2-MZ precursors (230), and MZ B cells (231).

Of note, the surface markers used to characterize the identity of the IL-10^+^ B cells change following activation and might be not suitable to define a specific B cell subtype under inflammatory conditions. Interestingly in this context, it has been shown that “B10” cells upregulate the expression of the transcription factors Blimp1 and IRF4 while downregulating that of Pax5, suggesting that these cells undergo plasma cell differentiation. Moreover, upon transfer into recipient mice, “B10” cells become antibody-secreting cells (232). More recently, CD138^hi^ plasmablasts in murine spleen (98) or lymph nodes (153) were described as the major producer of anti-inflammatory IL-10 and IL-35 *in vivo* with the ability to limit EAE. In accordance with these findings, we found that IL-10^+^ plasma cells exhibit profound anti-inflammatory activities in a model of EBA, a rare autoimmune skin disease (85). These cells induce IL-10 expression but reduce IFN-γ production in CD4 T cells, promote IL-10 production by CD4^+^/Foxp3^+^ Tregs and suppress neutrophil functions. Hence, IL-10^+^ plasmablasts/plasma cells represent an important anti-inflammatory B cell subtype.

Identification of the identity of IL-10^+^ B lineage cells may help to develop a novel method to induce these cells in a therapeutic setting. Stimulation of B cells with CpG-oligonucleotides induces both plasma cell differentiation and IL-10 expression. Accordingly, experimental induction of IL-10^+^ B lineage cells by adaptive transfer of CpG-stimulated B cells has recently been shown to suppress ongoing EAE inflammation in a therapeutic setting (200). This approach may open a novel perspective for the treatment of inflammatory autoimmune diseases.

## Concluding Remarks

The success of current B cell targeting therapies emphasizes the important roles B cells play in the pathogenesis of autoimmune diseases. There is overwhelming evidence from animal models indicating that B lineage cells exhibit multiple powerful pro- and anti-inflammatory capacities. The current experience with B cell targeting therapies suggests that these findings also hold true in the clinic. Hence, therapies that specifically deplete pathogenic B cells and plasma cells, or generate immunosuppressive B cells/plasma cells could hold great potential for the treatment of autoimmune diseases. In an optimal setting, the therapy would be tailored to the individual patient based on his/her predicted needs, benefits, and risks.

## Author Contributions

KH, A-KC, and RM contributed to reviewing the current literature and writing of the manuscript.

## Conflict of Interest Statement

The authors declare that the research was conducted in the absence of any commercial or financial relationships that could be construed as a potential conflict of interest.
